# Societal attitude and behaviours towards women with disabilities in rural Nepal: pregnancy, childbirth and motherhood

**DOI:** 10.1186/s12884-019-2171-4

**Published:** 2019-01-09

**Authors:** Hridaya R. Devkota, Maria Kett, Nora Groce

**Affiliations:** 1 University College London (UCL), 1 – 19 Torrington Place, London, WC1E 6BT UK; 2 Leonard Cheshire Research Centre, Department of Epidemiology and Public Health, University College London (UCL), London, UK; 3 Leonard Cheshire Research Centre, University College London (UCL), London, UK

**Keywords:** Societal attitude, Disability, Pregnancy, Motherhood, Nepal

## Abstract

**Background:**

This study reviews the attitudes and behaviours in rural Nepalese society towards women with disabilities, their pregnancy, childbirth and motherhood. Society often perceives people with disabilities as different from the norm, and women with disabilities are frequently considered to be doubly discriminated against. Studies show that negative perceptions held in many societies undervalue women with disabilities and that there is discomfort with questions of their control over pregnancy, childbirth and motherhood, thus limiting their sexual and reproductive rights. Public attitudes towards women with disabilities have a significant impact on their life experiences, opportunities and help-seeking behaviours. Numerous studies in the global literature concentrate on attitudes towards persons with disabilities, however there have been few studies in Nepal and fewer still specifically on women.

**Methods:**

A qualitative approach, with six focus group discussions among Dalit and non-Dalit women without disabilities and female community health volunteers on their views and understandings about sexual and reproductive health among women with disabilities, and 17 face-to-face semi-structured interviews with women with physical and sensory disabilities who have had the experience of pregnancy and childbirth was conducted in Rupandehi district in 2015. Interviews were audio-recorded, transcribed, and translated into English before being analysed thematically.

**Results:**

The study found negative societal attitudes with misconceptions about disability based on negative stereotyping and a prejudiced social environment. Issues around the marriage of women with disabilities, their ability to conceive, give birth and safely raise a child were prime concerns identified by the non-disabled study participants. Moreover, many participants with and without disabilities reported anxieties and fears that a disabled woman’s impairment, no matter what type of impairment, would be transmitted to her baby, Participants – both disabled and non-disabled, reported that pregnancy and childbirth of women with disabilities were often viewed as an additional burden for the family and society. Insufficient public knowledge about disability leading to inaccurate blanket assumptions resulted in discrimination, rejection, exclusion and violence against women with disabilities inside and outside their homes. Stigma, stereotyping and prejudice among non-disabled people resulted to exclusion, discrimination and rejection of women with disabilities. Myths, folklore and misconceptions in culture, tradition and religion about disability were found to be deeply rooted and often cited as the basis for individual beliefs and attitudes.

**Conclusion:**

Women with disabilities face significant challenges from family and society in every sphere of their reproductive lives including pregnancy, childbirth and motherhood. There is a need for social policy to raise public awareness and for improved advocacy to mitigate misconception about disability and promote disabled women’s sexual and reproductive rights.

## Background

The UN Convention on the Rights of Persons with Disabilities (UNCRPD) Article 1 defines disability as the result of long-term physical, mental, intellectual or sensory impairment, which in interaction with various barriers, restricts the individual’s ability to participate in society on an equal basis with others. Disability is not the impairment itself but rather the product of attitudinal and environmental barriers [1].

WHO estimates that 15% of the global population has a disability. A higher prevalence of disability is reported among women in poor families in low-income countries [1]. The UNCRPD guarantees the sexual and reproductive rights of people with disabilities including their right to marry and have a family [2]. However, women with disabilities are too often prevented from enjoying these rights in many countries, including Nepal [3–5].

The literature suggests that society continues to undervalue women with disabilities, restricting their fundamental rights, including their sexual and reproductive rights and contributing to exclusionary practices by national governments, policymakers, and civil society. While women with disabilities have the same desire and legitimate right to become mothers as all other women, their childbearing and parenting ability is often brought into question [6, 7].

Nepal ratified the UN CRPD in 2010 [8]. In addition, there is a range of national laws and policies addressing the needs and rights of persons with disabilities [9], however, many people with disabilities still experience discrimination, denial of their rights and unequal access to basic services [5, 10].

Compounding this, patriarchal societies, such as that found in Nepal, have a strong gender bias favouring men. It is much harder for women with disabilities than their disabled male counterparts to engage in activities such as education, marriage, employment and political participation [11, 12].

Marriage is an expected cultural practice in Nepalese society, however, studies reveal that it is challenging for women with disabilities to find a marriage partner due to societal misconceptions and assumptions that incorrectly see such women are burdens rather than contributors to families and society [6, 11, 13, 14].

### Stigma, stereotype and prejudice

The terms ‘stigma’ and associated social responses such as prejudice and discrimination are often used interchangeably in the literature. Goffman [15] identified stigma as a feature that discredits and makes the person experiencing it different from others. This phenomenon is often accompanied by negative stereotyping, rejection, loss of status and discrimination [16]. A number of factors such as lack of knowledge, superstition, belief systems and fear contribute to stigmatization leading to exclusion of people with disabilities.

In Nepal, as in many cultures, disability has a long history of being perceived negatively as a misfortune caused by the curse of God, or associated with sins in a past life [17–19]. The negative social attitudes and behaviours towards disability are expressed in a number of ways including the exclusion of persons with disabilities from social roles and activities [20]. Thus, people with disabilities are less likely to have access to education, employment, marriage or to be allowed to participate in political and social events. Feeling uncomfortable with people with disabilities, avoidance and maltreatment are reported as are other forms of the negative attitudes and behaviours [20–22].

Evidence also shows that attitudes towards disability may change over time and differ from person to person and culture to culture [20, 21]. Attitudes also differ by type of disability, with those with more visible disabilities often facing greater discrimination and exclusion [14, 20, 23].

In this paper, we share findings from a study that focused on public beliefs and attitudes towards disability in rural Nepal with particular reference to the experiences of women with disabilities around sexual and reproductive health, specifically during their pregnancy, childbirth and motherhood.

## Methods

The study was conducted in Rupandehi, a southern district of Nepal with a population of 880,196 of which 50.89% are female [24]. Out of 125 recorded ethnic and indigenous groups in Nepal, the study district population is comprised of upwards of 95 different groups and indigenous inhabitants including 28 sub-groups of Dalits, who are grouped together as a socially and economically disadvantaged caste group and considered untouchables. The majority of people (78%) live in rural villages, though the urban population is growing fast. In terms of caste breakdown by the 2011 census, the study district population was comprised of 25% Janajati (indigenous), 21% Brahmin and Chhetries and 12% Dalit. 1.12% of the district population are reported to have a disability [24]. The Nepal Human Development Report 2014 reported life expectancy at birth for Nepalese people at 70 years. The national Human Development Indicator (HDI) value is 0.541, while the HDI value for the study district is 0.498 [25].

The data reported in this paper is extracted from a larger, original study that the authors conducted to investigate maternal healthcare access for disabled and Dalit women in Nepal. The larger study followed the mixed-methods approach by which quantitative and qualitative data were collected simultaneously. The study collected quantitative data using a survey questionnaire while qualitative in-depth interviews and focus group discussions were conducted to understand the experience of disabled study participants, non-disabled women with a range of social and educational backgrounds from the same communities and the views towards disability of women who serve as Community Health Volunteers. This paper reports the findings from a sub-sample of the qualitative component of this larger study.

Women with disabilities, Dalit and non-Dalit women without disabilities and Female Community Health Volunteers participated in this study. Face-to face semi-structured interviews with 17 women with physical and sensory disabilities and six focus group discussions with women without disabilities in the study district were conducted to ascertain community attitudes towards women with disabilities. Four of the six focus groups were comprised of non-disabled women from the surrounding community selected to represent a range of different ethnic backgrounds and educational levels. The total number of this group was 42 – with groups ranging in size from 10 to 12. An additional two groups of Female Community Health Volunteers, comprising 6 and 8 participants respectively, were chosen with the help of local health facilities.

All participants were purposively selected, and the interviews and discussions were conducted in a natural setting. With the help of local non-government organizations (NGOs) and disabled people’s organizations (DPOs) we sampled 19 women with physical, visual, intellectual, speech and hearing disabilities who had experienced pregnancy and childbirth. Two women, one with an intellectual disability and one with a hearing and speech disability were excluded from the interview because of the complexities involved in communication and in assessing mental disability due to the limited knowledge of the study team. An adapted screening tool from the UN Washington Group on Disability Statistics (short set) was used for disability assessment [26, 27]. Interviews with women with disabilities were conducted individually in their homes.

The focus group discussions were conducted in four different villages with diverse groups to capture the views from multiple perspectives. To reflect the key social divisions within the area, both Dalit and non-Dalit women (two groups each) were included in the discussions. Dalit are considered ‘untouchables’ and are at the bottom of the caste hierarchy, constituting about 12% of the district population [28]. Two additional focus group discussions were conducted with the Female Community Health Volunteers to understand their service experience and views towards pregnancy and childbirth in women with disabilities. This was important as they play a key role in delivering basic maternal-child healthcare serve as the first contact at the community level. The number of interviews and focus group discussions were determined by data saturation.

Interview checklists and topic guides were used in conducting in-depth interviews and focus group discussions. The checklists and topic guide for focus group discussions covered participant’s beliefs and values concerning disability; views on sexual and reproductive needs and marriage of women with disabilities (with particular focus on pregnancy and childbirth among women with disabilities); and their feelings and levels of comfort around women with disabilities. The interview guide for women with disabilities included questions on their own views and experiences in the family, society and workplace in regards to their disability, marriage, pregnancy and childbirth. The checklist and topic guides were field-tested and the first author, a native Nepali speaker, with the help of two local trained female research assistants, conducted the discussions and interviews.

The role of the research assistant was to obtain consent of participants and to take notes during interviews and discussions. Developing a sustained contact, we fostered a relationship with study participants and encouraged their contribution. Considerable effort was put into maintaining neutrality and balancing the power relationship between the researcher and the participants at all stages of the research process. All interviews and discussions were audio-recorded with the participant’s written approval.

After completion of field data collection, we followed a series of steps before the analysis proceeded to the interpretive phase. The first step involved transcribing verbatim all the audio-recordings in Nepali and translation into English which was done by the first author and three other language specialist. Then the first author reviewed all transcripts and the interview notes, reading, rereading and reviewing for overall understanding. Following the framework method developed by Ritchie and Spenser, we then analysed data in five stages: familiarization; identifying a thematic framework; indexing; charting/mapping; and interpretation [29]. To ensure accuracy for inter-rater reliability, a second person, the senior Project Coordinator of the larger study, assisted in conducting the interviews, crosschecked the transcriptions, translations and data coding. At this stage, where no new concepts emerged from the further review and coding of data, we developed sub-themes and grouped together the concepts identified in the text based on their similarities and relationships to develop themes and subthemes (Table 1). The themes and subthemes were then analyzed in relation to the research questions and are described in the following section.

**Table 1:** Themes and Sub-themes

Theme	Sub-theme
Misconception and misunderstanding about disability	- Disability as a symbol of misfortune
	- Doubts about sexuality and ability to conceive and care
	- Passing on their disability
	- Disability as their identity
Neglected/Ignored sexual and reproductive needs and rights of women with disabilities	- Discouragement of disabled women’s marriage and family life
	- Pregnancy and child birth of women with disability as an additional burden to the family and society
Rejection and Exclusion by the family and society	- Rejection of reproductive choice
	- Exclusion from family decision making and community groups
Facing challenges due to powerlessness	- Discrimination and exploitation
	- Violence and abuse
	- Perceived risk and fear
Emotional support	- Encouragement from family, neighbours and healthcare providers
	- Empathy, love and support; strengths

## Results

### Characteristics of study participants

The sampled study participants consisted of 12 women with physical disability, four with visual disability and one with a hearing and speech disability. The majority [12] of the participants were non-Dalit while five were Dalits. The ages of these women ranged between 23 and 35 years. All women were married and had personal experience of pregnancy and childbirth. Less than half (seven) reported that they found partners themselves. Over one-third of women had no formal education while four women had some college education.

### Misconception and misunderstanding about disability

Participants who had disabilities reported that their disabilities were regularly regarded by others as a misfortune, and they frequently encountered inappropriate behaviour from neighbours and society. Women with disabilities reported being regularly humiliated, stigmatized and negatively stereotyped.

A woman with a physical disability expressed her frustration about how the community treats her due to their misconceptions about disability:

*If somebody is going out and meets a person with disabilities, they say – it is bad luck, I saw the face of a disabled….. We are blamed if they are unsuccessful in work; this is the kind of discrimination we are facing. If we participate in any ceremonies and weddings, they say, ‘Why did she come here? Everybody will see her and some bad things may happen.*

***- A Dalit woman with physical disabilities***

Another woman with a visual disability stated:

*There was an incident during my first baby. It was during “Teej festival” (Festival of Women) when I had gone to a fair. My baby was three and half months old. A woman there said that it was pathetic to see a blind person having children. I did not recognize the woman, but I got very angry. Why did I have to be a character of sympathy when everything was normal? Had the baby been in pain or had it been crying, such comment would be meaningful. I returned home without going around.*

***- A non-Dalit woman with visual disabilities***

Participants from disabled and non-disabled focus groups reported that folk beliefs about the sexual desires and reproductive capability of women with disabilities persist and that their sexual well-being is often neglected. In Nepali culture, women do not openly talk about sex and sexuality; however, as non-disabled focus groups were ‘female-only’, discussions about these topics was more open. The participants in focus group discussions, none of whom had disabilities, stated that due to cultural and social mores their families and neighbours regularly spoke negatively about sexual desire and ability to conceive for women with disabilities. Only one non-disabled focus group participant raised the issues of rights and argued that people with disabilities have the right to have children. Many focus group participants agreed that people with disabilities have the same desires as people without disabilities. However, not everyone agreed. As one educated participant with a physical disability in her in-depth interview recalled, her own grandmother-in-law was suspicious about her ability to conceive:

*We had a grandmother here but it’s been about 2 years since she died; she used to keep on asking if I would have the baby so I guess she might have had that feeling. After 6 months of her dying, I became pregnant.*

***- A non-Dalit woman with physical disabilities***

When asked about adult relationships and intimacy, almost all the focus group discussion participants without disabilities stated that women with disabilities can have relationships, become pregnant and give birth, but that they would not be capable (had no ability) of caring for and rearing a baby. Some of the women with disabilities reported that their parents did not understand their emotional and sexual needs and never talked to them about marriage.

Many of the focus group discussion participants believed that emotions and desires about sexuality and pregnancy for women with disabilities are the same as for women without disabilities. As two of the focus group discussants noted:

*I think that the desire for sexuality is the same for people with disabilities and people without disabilities but there are differences in problems and difficulties.*

***- FGD/non-Dalit Women***

*…..of course they want to have a baby. Every woman wants to have a baby. People think that after having a baby, it will grow up and support. He will earn and feed the family later.*

***- (FGD/Dalit Women***

Other focus group participants reported that people in the community have both positive and negative views towards pregnancy, childbirth and motherhood for women with disabilities:

*All people will not have the similar thoughts; some views in a negative sense and disgust; some say that she needs help for herself and how she rears the baby and some others show their sympathy.*

***- FGD/Non-Dalit women***

In a focus group discussion of female community health volunteers, one woman added an additional cultural interpretation. The meaning of giving birth, she said, for a mother is to be satisfied with all senses. If a mother cannot see the baby, hear the baby cry or play with them, then what would be the point of having a baby:

*If they are blind, then it will be difficult. If they give birth, there will be a problem. Who will take care of the child? If they cannot hear the baby’s cry, then what is the meaning of giving birth? It will be really difficult…*

***- FGD/Female Community Health Volunteers***

One participant with visual disabilities expressed her disappointment that even after demonstrating her ability doing all household chores, some family and neighbours doubted her ability to care for a baby:

*I did hear such comments and doubt on how I would take care of a baby when I myself could not see. But they had seen me doing all the household chores. So people had mixed opinion; some said I would take proper care whereas the others said I would not.*

***- A non-Dalit woman with visual disabilities***

Another widely held belief is that a mother’s disability will usually be passed on to her baby***.*** This was found to be a primary reason for negative attitudes among people without disabilities towards marriage and pregnancy in women with disabilities. Women with disabilities were often counselled not to marry or were not considered acceptable marriage partners because of this misconception. Some FGD participants firmly believed that the baby and subsequent generations would inherit any disability present in the mother, others disagreed. Few participants, however, demonstrated any knowledge of the fact that some types of disabilities were congenital and many others were not. As one participant noted:

*They should not give birth. The baby might also have a disability due to the disability of mother, so it is risky.*

***- FGD/Dalit Women***

One participant with visual disabilities expressed her frustration that this belief often discourages her from becoming pregnant:

*People say disability is often hereditary. Since both of us were blind, everyone thought our life would be complicated with a baby. Some of the neighbours said we should not have planned for a baby and most suggested it would have been better if we had used family planning devices. I used to say to my neighbours that not all disability is hereditary; some could be and some not; whatever happens we will see….*

***- A non-Dalit woman with visual disabilities***

The study participants reported that disability is the concern of the whole family, with society stigmatizing non-disabled family members as well and that this often complicates their own marriages and relationships. As one participant with visual disabilities stated:

*When there is a person with disability at home, everything gets connected to him/her. For example, I am a blind person in my home, so when my elder brother was getting married, the issue of looking after me was raised by many. Also, people tend to think the baby to be born in the house will also be blind, people think it is heredity…. People often looked at the eyes of my brother’s children; so it is obvious that they would talk about our baby.*

***- A non-Dalit woman with visual disabilities***

Societal and cultural beliefs exert a strong influence upon individuals, creating doubts and fears even if the individual is educated. For example, a well-educated participant with physical disabilities who did not initially believe her disability would be inherited, later developed doubts after talking to her neighbours:

*I had a fear that my baby would have the same disabilities as me when I heard things from the society. Because of the belief that we have in society, I had doubts in my mind.*

***- A non-Dalit woman with physical disabilities***

Some Dalit women without disabilities in the focus group discussion argued that all babies born to parents with disabilities do not acquire disability:

*…. they may have normal children. There are examples that the deaf have very clever children. Both the mother and father are deaf but their children are talent. In some cases, there could be heredity.*

***- FGD/non-Dalit Women***

Negative attitudes were also expressed in relation to identity. Many of the women with disabilities reported that on many occasions as a child, they were not given a name at all, but just referred to as their disability (i.e. the blind girl, and lame one). In the eyes of others, their identity was their disability. Many reported that they found this humiliating and an assault on their individual identity.

### Neglected or ignored sexual and reproductive needs and the rights of women with disabilities

Marriage between people with and without disabilities was often not easy. The study participants – both non-disabled and disabled - reported that marriage of a woman with disabilities is a complex issue. Factors include benevolent protection from parents who fear that another family would not treat their daughter properly; fear from the paternal family that the woman with disabilities would not be “good enough” for their son and would prompt malicious gossip; fear about conception, childcare and domestic responsibilities. Some FGD participants expressed the view that people with disabilities should be paired off with other people with disabilities.

Interestingly, the majority of the women with disabilities interviewed were married to male partners with disabilities. In addition, most of them had chosen their partner, as opposed to having an arranged marriage. This was in stark contrast to the social practices in the study area, where arranged marriages remain the norm. These participants reported that their families had not considered arranging a marriage for them; therefore, they had sought a partner of their own and lived separately from the extended family.

A smaller number of the participants with disabilities reported that their family members were positive and helpful about their marriage and pregnancy. A woman with visual disabilities reported that her mother-in-law and other family members regularly reassured her, saying that her husband with visual disabilities would be able to create a happy life for them:

*Even my mother-in-law used to say that my husband would keep me happy no matter what, so she often told me not to worry. Even my great-mother-in-law was supportive and so were other family members.*

***- A non-Dalit woman with visual disabilities***

A few of the study participants with disabilities were women who had married a man without disabilities. They reported that their partners had married them expecting to acquire their parent’s property as part of the dowry, which was an incentive for the marriage. However, they reported that these arrangements had not often succeeded, with further disputes concerning terms of the inheritance between the families, and subsequent breakdown of the relationship in many cases. One of the participants with disabilities, whose parents had bequeathed their property to her and who had married a man without disabilities described her experience:

*My husband had been asking for this property to convert to his name but I didn’t agree. Then he started torturing me. I could not live together with him and I was separated. It has been around 2 to 3 years now since we separated.*

***- A non-Dalit woman with physical disabilities***

The study found that many families and neighbours perceived pregnancy and childbirth in a woman with disabilities as an additional burden:

*It would be difficult if a woman with mobility problem (disabilities) gives birth. In such cases, it is better not to give birth. If the woman cannot take care of the baby, it would be difficult to those for giving birth as well. They will also have difficulty to care and rear the child. If she is blind or only the mobility disabled, she should give birth even for her own future support. It would be better to give birth as per the individual’s physical ability.*

***- FGD/Non-Dalit women***

*It would be as per the situation. Some love them and care more. But if they have given birth even with their severe type of disability, then the family or neighbours may perhaps look negatively and may feel disgust.*

***- FGD/Non-Dalit women***

It was found that women with disabilities faced enormous pressure from society’s negative attitudes about their pregnancy and childbirth. On many occasions, women with disabilities being interviewed for this project stated that they themselves felt guilty and a burden, and faced discouragement in all aspects of life. Many respondents with disabilities reported that their family, particularly their mother-in-law, was not helpful during their pregnancies. However, after the baby was born, mothers reported that most mothers-in-laws welcomed their new grandchild:

*Relatives and society view us as a burden to them and they think they have to look after us throughout their life. This opinion is prevalent in every person of the society. They think a blind person is incapable of doing every kind of thing. Maybe some people with visual disabilities do not get married because they do not want to. Nevertheless, people think they did not get married because of their blindness, nobody understands that even blind people have choices in life. Such things make us feel really bad.*

***- A non-Dalit woman with visual disabilities***

Another Dalit participant with physical disabilities stated that she often came across negative reactions from her neighbours. She would not be invited to neighbour’s functions, as they considered her disability a burden, saying:

*…..why invite people with disabilities to the ceremony, instead of getting help from them. We have to care for them…they cannot do anything….they come, sit and only talk ……they are not helpful….*

***- A Dalit woman with physical disabilities***

The same participant recalled that she faced more trouble from her family than from the neighbours during her pregnancy and childbirth. She reported that her mother-in-law was negative and totally unhelpful when she was pregnant, so much so that her husband brought her back to her own parents’ home for the delivery:

*Other family members said, ‘We should feed her and take care of her child too, let her stay there.’ My mother-in-law said,’ If I had given birth to you, I would care for you’, so I stayed 5–6 months with my mother. Nobody came from my husband’s family to bring me back from my maternal home. When my baby started to crawl, my husband came to bring me back, without the permission of his mother. My mother said, ‘I will not send my daughter if you cannot take care of her. I will care for her whatever I can.’*

She further described the fact that her sister and mother were supportive, cared for and counselled her, keeping her with them during her pregnancy and childbirth, while she was being badly treated by her mother-in-law:

*My mother…she tried to convince me that many people (who have disability) do not get married, but you are lucky so you got married…. who could have known that your new family members would not care for you after marriage……. Sometimes I thought to commit suicide by taking poison even after conceiving.*

***- A Dalit woman with physical disabilities***

In Nepal, mothers-in-law have a powerful influence over their sons’ attitudes. As the woman above continued:

*I felt bad…I had given birth to a child that had added more trouble…I was tolerating the rudeness and bad behaviour while I was alone….but after having the baby, I had the additional responsibility to care for the baby. Nobody would marry me as well….I had pain and became restless by thinking all this. Somebody had talked to my husband so he came to take me back with him.*

***- A Dalit woman with physical disabilities***

Some participants said that having a child was part of a strategy to ensure future support for people with disabilities as a parent.

### Rejection and exclusion by the family and society

As noted above, the study found that families of women with disabilities in the study population commonly denied the rights of women with disabilities to marry or have children in the first place. The reasons included family prestige, over-protection by the parents, lack of understanding about disability and the reproductive needs of people with disabilities, and misconceptions created by stereotyping and prejudice, including around the fear of inheritance of the disability, as noted earlier.

A Dalit woman with physical disabilities stated that her husband was blamed for marrying her and excluded for several years by his parents and relatives:

*They did not talk to me and my husband for a year. They had scolded so much saying he should have searched (for) a non-disabled woman, why did he marry me….. they said I cannot plant paddy, cannot do other works, why he married with such a woman? They did not speak for a year with him too. Later they said to him that ‘It was your fate, you did not follow what I said but married such (a person)’. But earlier they used to scorn us saying he would not have a child by marrying a woman with disabilities.*

***- A Dalit woman with physical disabilities***

Another participant with visual disabilities had a similar story. She chose her partner with visual disabilities herself and their marriage was initially rejected by the husband’s family until it was clear the child had not inherited their blindness:

*With the first child, the problem was that we had not been accepted by our home/ family as we got married ourselves. Moreover, people thought that our babies would also be blind. Only when they realized that the baby could see, then only was I taken home along with the baby. They bought a separate home in Bhairahawa and kept us there. Now it is different, we have very good relation with other family members. Earlier it was very difficult.*

***- A non-Dalit woman with visual disabilities***

Women with disabilities were asked about their involvement in major family decisions and attendance at neighbour’s functions to understand their inclusion within as well as beyond the family. Few participants reported involvement in their family decision-making. The majority of respondents with disabilities also reported that they were not involved in the women’s groups. Some who had been part of women’s groups reported that they felt discriminated against, disdained or considered inferior, prompting many to leave such groups. One participant reported that the group specifically doubted her ability to make monthly savings contributions and did not invite her to become a member:

*What should I say why they don’t call when the neighbours go there. That is why I don’t feel like going there and I will not go there…They might have the thought ‘How will I get money to be in the group’.*

***- A non-Dalit woman with physical disabilities***

It was also apparent that many communities excluded women with disabilities from participating in ceremonies and rituals, considering their presence bad luck. One of the participants reported:

*Some people say it is unfortunate if they see us; some do not like us to be present in ceremonies and rituals considering us as a symbol of bad luck. If I go somewhere and anyone comments negatively, I do not go again. I have heard somebody saying She came herself in spite of sending other family members.*

***- A Dalit woman with physical disabilities***

### Facing challenges due to powerlessness

Some of the FGD participants without disabilities and many of the participants with disabilities in their in-depth interviews reported that women with disabilities are discriminated against in every sphere of life. Some participants with disabilities reported that Female Community Health Volunteers do not visit them, while women without disabilities are visited and counselled during their pregnancy. A few participants reported that whilst initially invited to attend women’s group meetings, they subsequently felt ignored and their opinions disrespected, prompting them to leave the group.

Importantly, women with disabilities further stated that the discrimination is not only outside but is also within their homes. One participant with a disability described the discrimination she faced from her own family members during her pregnancy and childbirth:

*There was so much….I am afraid to talk with anyone about those times, and the discrimination and troubles that I faced. I have to reassure myself and I like to take satisfaction because of my children. Both of us, me and my sister-in-law, delivered at home. Nobody helped me but the entire family cared throughout the 24 h while my sister-in-law gave birth. I was at my maternal home when I gave birth to my son and had good food, but with my daughter, they gave me cheap food.*

***- A Dalit woman with physical disabilities***

Another Dalit woman with physical disabilities reported that she was discriminated against at work by the neighbours due to her disability. She stated that her mother also frequently abused and discriminated against her before her marriage. Her mother continues to do so as she lives close by:

*There are two younger sisters, they love me but mother hates me. They are far away, so mother loves them. I am disabled and she does not love me! My leg became weak and my mother used to verbally abuse me; she said that it would be better if I had died.*

***- A Dalit woman with physical disabilities***

An FGD participant described discrimination and exploitation within her own family to a niece with hearing disabilities:

*I have a niece who cannot speak well, she got married but people at her home didn’t care for her. They thought deaf people should be given leftover food, as she cannot speak for herself. Such is the perspective of people.*

***- FGD/Female Community Health Volunteers***

The study found that people in the society think that women with disabilities are weak and have no power. Such an environment creates feelings of helplessness and fear in the minds of women with disabilities. The participants reported many examples of violence, abuse and exploitation by the family members. As one of the study participants noted:

*Sometimes I had such feeling. I felt as weak, not able to do anything. Even when people said something good, I felt they were saying it to humiliate.*

***- A non-Dalit woman with visual disabilities***

Both Dalit and non-Dalit women with disabilities reported facing challenges in the family and society due to their disability. However, Dalit women with disabilities stated their experience of disparities, exclusion and bad treatments in the society was due more to their disability rather than their lower caste status. A Dalit participant with disabilities expressed her dissatisfaction at being stigmatized and mistreated:

*Being disabled is more painful….If I did not have a disability nobody would speak bad or painful words to me…I would not seek support or help from anybody….society would not consider me a symbol of bad luck and I would not be excluded.*

***- A Dalit woman with physical disabilities***

Some of the respondents reported that their husband or other family members abused them. One participant reported that her mother frequently abused her verbally and physically due to her disability.

*…….helped by my brother-in-law. He has known all about me and my trouble, how I was suffering being scolded and beaten. I could do work and was also doing, but she (mother) used to beat me saying that I was sitting idly and eating, doing nothing.*

***- A Dalit woman with physical disabilities***

### Emotional support

Not all respondents reported negative attitudes to women with disabilities. Despite the negative social environment, a number of participants, both in focus group discussions and individual interviews reported that their families and neighbours were supportive and positive toward disabled people. Some disabled women specifically reported that their neighbours were kind, sympathetic and supportive during their pregnancy and encouraged them to go for services. Some also reported that Female Community Health Volunteers visited them at home during their pregnancy.

As one of the FGD participants stated:

*All people will not have the similar thoughts; some views in a negative sense and disgust; some say that she needs help for herself and how she rears the baby and some others show their sympathy.*

***- FGD/Non-Dalit women***

## Discussion

Findings from this study provide a range of insights from both women with disabilities themselves and from members of the families and communities in which they live. It is interesting to note that the culture and social attitudes towards women with disabilities was often reported as unfavorable, with misconceptions about disability in general indicating that negative social attitude towards disability prevailed in the study district. Findings revealed that many women with disabilities are stigmatized and discriminated against in various forms by society and even within their own families. However, importantly, while exclusion and negative attitudes were commonly reported by and about women with disabilities, the findings were mixed with some women with disabilities as well as some people without disabilities expressing attitudes that are more inclusive.

In relation to the negative attitudes and social behaviours towards women with disabilities, several key issues were identified and despite many people’s openly prejudiced views, some degree of “benevolent prejudice” towards pregnant women with disabilities was also common. Issues regularly raised in FGD and interviews included the marriage of women with disabilities, their ability to conceive, give birth and safely raise a baby. Moreover, many respondents with and without disabilities reported anxieties and fears that their impairment would be transmitted to their babies and that pregnancy and childbirth of women with disabilities would be an additional burden for their family.

The study found little exposure to, and insufficient knowledge about disability among participants without disabilities, leading to blanket assertions, which resulted in discrimination, rejection and exclusion of people with disabilities. Many women with disabilities reported that they faced discrimination and humiliation as well as violence from their family members, particularly from their mothers-in-law and husbands. The study reflected more broadly, already established findings that women with disabilities live under various forms of oppression, which includes being denied opportunities and facing rejection, showing that women with disabilities are often not valued in Nepalese society and sometimes have no individual identity beyond that of their disability.

As in other societies around the world, myths, folklore and misconceptions about disability such as ‘disabled people are tragic figures that society should pity’ [30–33], were found commonly among the individuals without disabilities and community health worker groups interviewed. Consistent with this finding, the literature also shows that the negative attitudes more commonly exist among poor and education-poor communities [33–36].

Beliefs about disability expressed by the non-disabled participants in this study are also commonly found in the religious and folk beliefs in many traditions including Hinduism, Buddhism and Islam. For example, in India and Nepal, many people believe that disability is a punishment or curse from God. Moreover, people with disabilities are traditionally perceived as inauspicious and are often discouraged from attending religious and wedding functions [19, 33, 34]. While Hinduism has as a central tenet the concept of equality, the strong belief in reincarnation is sometimes interpreted to mean that people disabled in this life may have done something wrong in a previous life [19, 33, 37].

Exclusion was often expressed through patronising attitudes. These were often manifested through people in the community questioning the ability of women with disabilities to exercise their right to make key life decisions around marriage, pregnancy and childbirth. A number of factors such as inadequate knowledge about disability and the needs of people with disabilities, misconceptions and incorrect beliefs, as well as fear of contagion, the inheritance of disability and uncertainty about how to interact with people with disabilities, contributed to this negative attitude. The focus of this particular paper is the question of pregnancy, childbirth and motherhood among women with disabilities who already have one or more children. A linked but important additional question addressed elsewhere [5] is the access of women with disabilities to contraception and the availability of this access compared to that of their non-disabled peers.

Positive perceptions about the ability of women with disabilities to give birth and rear their children were minority views, however they did exist. And there was strong variation regarding these perceptions by disability type. For example, women with intellectual or mental disabilities were often presumed to pose a greater risk to the child than were women with other types of disability. The families routinely, although not universally, perceived the women with a disability as a burden since they assume this woman would contribute less to family chores and income. Such negative attitudes led to discrimination within families with little or no priority given to the needs of women with disabilities including their treatment, rehabilitation or other essential care required.

Issues related to the ability of women with disabilities to marry and doubts about their ability to give birth and rear children are consistently highlighted by studies conducted in countries such as India and Korea [33, 34, 38–40], however, not all research is consistently negative on this. In contrast to our findings, another Nepali study by Simkhada et al. [19] found positive attitudes towards the rights of women with disabilities to marry and have children. Such contradiction in people’s views is not surprising in a multi-cultural society like in Nepal. Moreover, this study looked at different groups with lower educational and awareness levels in a different part of the country than did Simkahada et al.

Significantly, women with disabilities themselves often shared reservations about their ability to successfully marry, become pregnant and raise children. While some had come to understand and appreciate their own ability and had some knowledge of new and changing attitudes regarding the rights and potential of women with disabilities, a number had not been reached by progressive ideas and attitudes regarding people with disabilities.

Evidence shows that negative attitudes towards disability are changing gradually [21]. This study reflected some of this changing attitude, with respondents reporting some positive attitudes towards people with disabilities, and respondents with disabilities reporting numerous examples of kindness and acceptance. Some of this is also based on individual attitudes and on familiarity with the disability from personal or family experiences. Whilst in this study disabled participants perceived these as positive experiences, it could be argued that these actions were more closely linked to paternalistic caring, rather than reflecting notions of equality and mutuality. However, increased education and levels of awareness among the public, changing socio-cultural contexts, and policy changes including Nepal’s ratification of the CRPD and the development and passage of a number of related laws and policies in line with the CRPD, might also be influencing changing public views about women with disabilities.

It is important to note that women with disabilities showed not only vulnerability but a number of strengths. For example, many who felt that their families were unwilling or unable to find them marriage partners had identified and arranged their own marriages, often in the face of considerable opposition. This self-starting approach to marriage, which flies in the face of established custom, is worth a more in-depth discussion than can be provided here, but it is of note. Many disabled women reported wanting a child, deciding to become pregnant and seeking antenatal health care as well as support for childbirth, even though they knew or feared that they would meet with resistance and lack of support by some family, health care providers and members of the surrounding community. There was also an understanding expressed by some women with disabilities and as well as members of the broader community that in a very practical sense, having a child represents long term planning as it guarantees that the disabled woman – as is true for many other women in the community – some security and support in older age.

At the outset of this study, we hypothesized that women who were both disabled and Dalit would be doubly discriminated against. This was based on studies that state women with multiple vulnerabilities may face compounded discriminations [41]. Significantly however, this study found disability far outweighed class as a daily concern. Disabled women, both Dalit and non-Dalit faced similar challenges. Dalit women with disabilities consistently reported that they experienced discrimination due to their disability rather than their lower caste status. Non-Dalit women reported facing barriers in education, social inclusion and family life very similar to those reported by Dalit women. Further studies are needed to explore this intersectional issue in greater depth.

Finally, it is important to note that there was a mix of attitudes throughout the community, based on a range of factors including membership in different ethnic/ minority groups, personal familiarity with disability, education and individual beliefs and temperament. This mix of attitudes in the public arena, even in a remote area, is an interesting finding and one that generates recommendations for policy and practice. There is certainly a need to encourage social policy and information efforts to raise public awareness and improved education and advocacy campaigns to mitigate against misconceptions about disability and promote the sexual and reproductive rights of women with disabilities. But the range of attitudes and beliefs found also offers an important starting point for such efforts – it may be possible to build on the best and most progressive attitudes towards women with disabilities already existing in the community. And such interventions must not only target the general community. Our findings show that many women with disabilities themselves need more information and support as they move forward through pregnancy, childbirth and motherhood.

We acknowledge several limitations associated with this study. The study was a part of a Safe Motherhood Project in Nepal, therefore, the study population was limited to one project district. Additionally, like all qualitative studies, our findings may not be generalizable to other areas with different social and cultural contexts. Furthermore, the views expressed by the participants reflect the attitudes towards disability in general rather than specific types of disability.

## Conclusion

Although negative attitudes are prevalent among the public in the study district towards women with disabilities, their marriage, pregnancy and motherhood, we found a range of attitudes related to pregnancy, childhood and motherhood among women in the general public in this area of Nepal. Without doubt, women with disabilities face significant challenges from family and society in every sphere of life due to negative attitudes which reflects inadequate public knowledge and misconceptions about disability, stereotyping and prejudice. Yet there were also a range of positive attitudes expressed by focus group members that warrants further exploration and that could provide a starting point for positive changes in policy and programmes to better support women with disabilities who become pregnant in this region. And finally, it is important to emphasize that disabled women themselves faced a number of significant social and economic challenges, but also showed a range of strengths that must be supported and encouraged.

## Abbreviations

DPO: Disabled People’s Organization
FCHV: Female Community Health Volunteer
FGD: Focus Group Discussion
NHRC: Nepal Health Research Council
UCL: University College London
UNCRPD: United Nation’s Convention on the Rights of Persons with Disabilities
